# Cinacalcet increases renal calcium excretion in PTHrP-mediated hypercalcemia: a case report

**DOI:** 10.1186/s12902-023-01386-3

**Published:** 2023-06-16

**Authors:** Samya Faiq, Kristen Lavelle, Tina Hu, Dolores Shoback, Gregory Ku

**Affiliations:** 1 School of Medicine, University of California Davis, Davis, USA; 2 Department of Medicine, Division of Endocrinology and Metabolism, University of California, San Francisco, 513 Parnassus Ave, HSW 1027, San Francisco, CA 94143 USA; 3 Department of Veterans Affairs, Endocrine Research Unit, San Francisco, CA USA

**Keywords:** Osteonecrosis, Bisphosphonate, Hypercalcemia, PTHrP, Cinacalcet, Case report

## Abstract

**Background:**

In the acute setting, PTH-independent hypercalcemia is typically treated with anti-resorptive agents such as zoledronic acid or denosumab. When these agents are no longer able to control hypercalcemia, several case reports have shown the utility of cinacalcet. However, it is not known if cinacalcet can be effective in patients naïve to anti-resorptive therapy or how cinacalcet ameliorates the hypercalcemia.

**Case presentation:**

A 47-year-old male with a history of alcohol-induced cirrhosis was admitted for left cheek bleeding and swelling from an infiltrative squamous cell carcinoma of the oral cavity. On admission, he was found to have an elevated albumin-corrected serum calcium of 13.6 mg/dL, a serum phosphorus of 2.2 mg/dL and an intact PTH of 6 pg/mL (normal 18–90) with a PTHrP of 8.1 pmol/L (normal < 4.3), consistent with PTHrP-dependent hypercalcemia. Aggressive intravenous saline hydration and subcutaneous salmon calcitonin were initiated, but his serum calcium remained elevated. Given tooth extractions scheduled for the next day and possible irradiation to the jaw in the near future, alternatives to antiresorptive therapy were sought. Cinacalcet was initiated at 30 mg twice daily then increased to 60 mg twice daily the following day. The albumin-corrected serum calcium level decreased from 13.2 to 10.9 mg/dL within 48 h. The fractional excretion of calcium increased from 3.7 to 7.0%.

**Conclusions:**

This case demonstrates the utility of cinacalcet for the treatment of PTHrP-mediated hypercalcemia without prior anti-resorptive therapy via increased renal clearance of calcium.

## Background

Following intravenous saline hydration, zoledronic acid or denosumab is the standard treatment for PTH-independent hypercalcemia [1, 2]. In contrast, calcimimetics such as cinacalcet and etelcalcetide are typically used to treat PTH-dependent hypercalcemia (e.g., primary and tertiary hyperparathyroidism) or to reduce PTH in secondary hyperparathyroidism [3, 4]. In both cases, calcimimetics are thought to reduce PTH secretion by parathyroid cells by activation of the calcium-sensing receptor (CaSR). If this is the sole mechanism of action, calcimimetics would seem to have little role in the treatment of PTH-independent hypercalcemia in which the PTH is already suppressed. However, animal studies show that calcimimetics can reduce the hypercalcemia induced by teriparatide, suggesting a role for calcimimetics in the treatment of PTH-independent hypercalcemia [5]. Furthermore, several case reports suggest that cinacalcet can be used in PTH-independent hypercalcemia that is refractory to antiresorptive therapy. However, the exact mechanism of action is unknown. We report a case of hypercalcemia due to increased PTHrP that responded well to cinacalcet therapy without previous bisphosphonate therapy and demonstrate that increased renal excretion of calcium may play a role.

## Case presentation

**Fig. 1:**
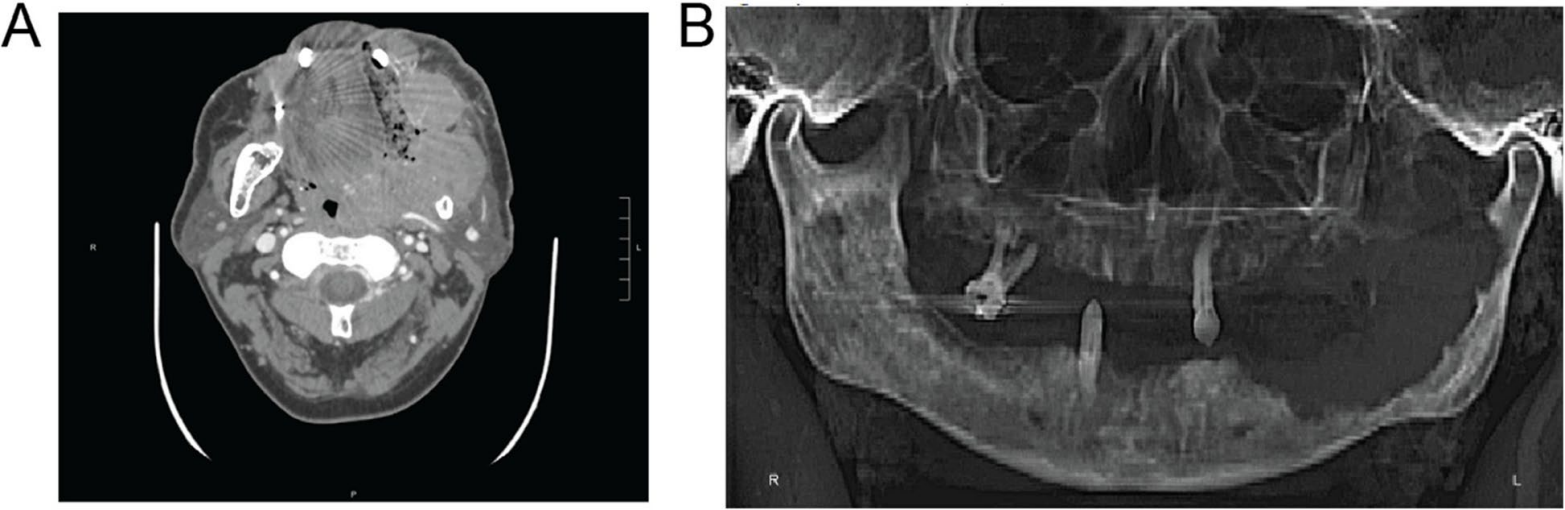
Imaging of a large squamous cell carcinoma of the oral cavity.** A** Cross sectional CT image demonstrating a 6.8 × 8.0 × 7.5 cm vascular soft tissue mass originating in the alveolar ridge of the left mandibular ramus with significant bony destruction. **B** X-ray prior to full mouth dental extraction showing 3 remaining teeth with gross caries and bony destruction of the left mandible

**Fig. 2:**
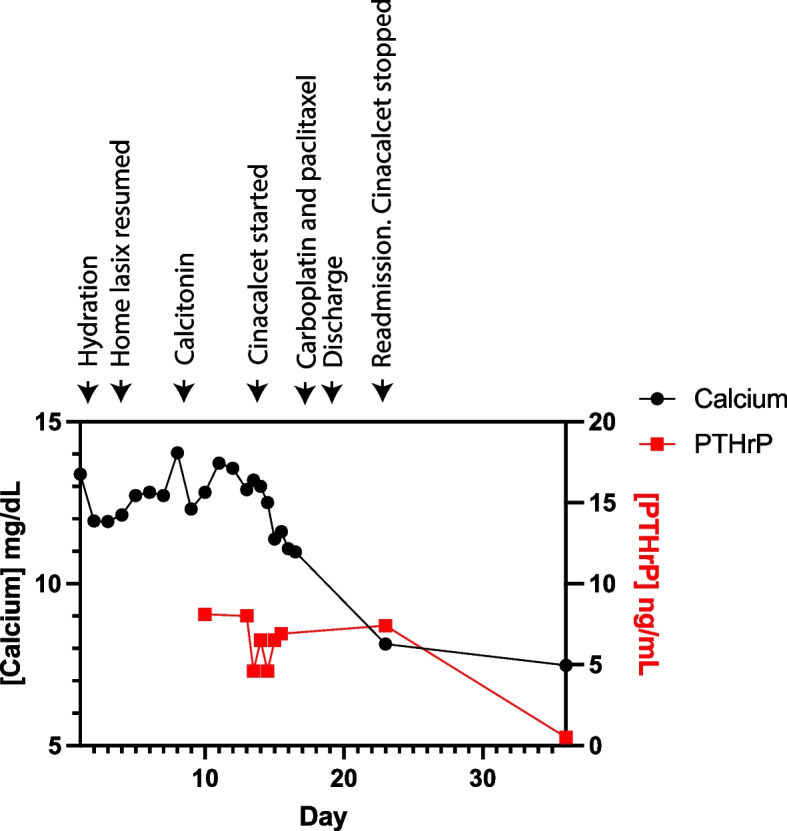
Serum calcium and PTHrP with major interventions. Left axis shows albumin-corrected serum calcium. Right axis shows PTHrP

A 47-year-old man with a history of alcoholic cirrhosis (Child-Pugh Class B) and esophageal varices with a history of ascites and lower extremity edema presented with recurrent left cheek swelling and bleeding. CT imaging showed a 6.8 × 8.0 × 7.5 cm vascular mass arising from the alveolar ridge of the left mandibular body with significant bony destruction (Fig. 1A). Biopsy of the lesion showed an infiltrative squamous cell carcinoma of the oral cavity, and chemotherapy with possible future irradiation was planned. However, the patient had only 3 remaining teeth; each was mobile and had gross caries (Fig. 1B). Given the risk for dental infection during chemotherapy, oral surgery recommended prophylactic extraction of his remaining teeth. On admission, he had an albumin-corrected serum calcium of 13.4 mg/dL. The serum phosphate was 2.1 mg/dL, PTH 6 ng/L(nl 18–90), PTHrP 8.1 pmol/L(nl < 4.3), 25-hydroxyvitamin D 22 ng/mL(nl 20–50), 1,25-dihydroxyvitamin D 11 pg/mL(nl 20–79), creatinine 1.18 mg/dL(nl 0.73–1.4), eGFRcr 77 mL/min/1.73m2(nl > 59), magnesium 1.2 mg/dL(nl 1.6–2.6), and serum sodium 130 mmol/L(nl 135–145). There was no lower extremity edema or evidence of ascites on exam or imaging. The patient was hydrated with intravenous isotonic fluids with improvement in albumin-corrected serum calcium to 12.0 mg/dL. After the biopsy, he was then restarted on his outpatient dose of furosemide at 40 mg orally per day (HD 4) for cirrhosis management and spironolactone 100 mg per day was restarted (HD 6); during this time, his urine output increased to a peak of 3.75 L/day and he was net negative 2.7 L. At that time, an abdominal ultrasound confirmed no ascites. His serum sodium increased to 136 mmol/L (nl 135–145). Unfortunately, his albumin-corrected calcium rose to 14 mg/dL and spironolactone was discontinued because of concern that he was becoming volume depleted. His ECG findings at peak albumin-corrected serum calcium revealed nonspecific ST and T wave abnormalities. He was given 3 doses of 4 u/kg calcitonin. His albumin-corrected serum calcium improved to 12.0 mg/dL but soon rose again back to 13.6 mg/dL (Fig. 2).

Endocrinology was consulted for persistent hypercalcemia. Symptoms included diarrhea and increasing confusion over several weeks but no constipation or abdominal pain. There was no family history of hypercalcemia and no personal history of multiple myeloma, other malignancy, granulomatous disease, excess use of calcium or vitamin D or A supplements, lithium, or thiazide diuretics. On physical exam, vital signs were normal. He was fully oriented but was tangential during the interview and somnolent. His oral cavity showed poor dentition, 3 remaining teeth, and a large mass in the right buccal space. He had mild asterixis. His abdomen was nondistended and he had no lower extremity edema. The hypercalcemia was thought to be due to PTHrP production from the oral squamous cell carcinoma.

Given the increased risk of osteonecrosis of the jaw with planned tooth extractions the next day and upcoming radiation to the jaw region, cinacalcet was started at 30 mg orally twice a day on hospital day (HD) 13 in an effort to avoid intravenous zoledronic acid or denosumab. The following day (HD 14), the dose was increased to 60 mg orally twice a day. The patient’s albumin-corrected serum calcium levels decreased from 13.2 mg/dL on the evening of HD 13 to 12.5 mg/dL on the evening of HD 14 after only 2 doses of 30 mg of cinacalcet. The fractional excretion of calcium increased from 3.7% prior to cinacalcet (HD 13) to 5.4% (HD 14). PTHrP declined to 4.6 pmol/L. PTH decreased to < 3 ng/L. Cinacalcet was increased to 60 mg twice a day on HD 15, and after two doses, his albumin-corrected calcium fell further to 11.6 with a PTHrP of 6.9 pmol/L. The fractional excretion of calcium increased to 7%.

The following morning (HD 16), his albumin-corrected calcium fell to 11.1 mg/dL. Carboplatin and paclitaxel were initiated on HD 16 to treat his squamous cell carcinoma, and he was discharged to home later that same day. Upon discharge on HD 16, his serum creatinine was 0.84 mg/dL, eGFRcr 109 mL/min/1.73m2, magnesium 1.8 mg/dL, and sodium 130 mmol/L. One week later, he was admitted to an outside hospital with sepsis, pancytopenia, an absolute neutrophil count of 30, hypomagnesemia, hypocalcemia (albumin-corrected calcium of 7.9 mg/dL), and diarrhea. The PTHrP remained elevated at 7.4 pmol/L. Cinacalcet was discontinued. The patient was discharged after empiric antibiotics and transfusion. Two weeks later, the albumin-corrected serum calcium fell further to 7.5 mg/dL, PTHrP was found to be 0.5 pmol/L(nl < 4.3) and PTH was 56 ng/L(nl 18–90), suggesting reduced tumor production of PTHrP in response to chemotherapy. Repeat CT imaging showed the tumor had shrunk substantially (5.6 × 2.4 × 5.1 cm). 25-hydroxy vitamin D levels were found to be low at 13 ng/mL, reduced from 22 ng/mL on the initial admission consistent with mild hypocalcemia caused by vitamin D deficiency and hypomagnesemia. Oral magnesium supplementation and cholecalciferol 4000 IU daily were started, and his albumin-corrected calcium rose to 9.3 mg/dL within one month. Radiation therapy versus pembrolizumab and cetuximab were recommended given his pancytopenia and sepsis after carboplatin and paclitaxel.

## Discussion and conclusions

We report a challenging case of PTHrP-mediated hypercalcemia of malignancy that was refractory to intravenous saline hydration and calcitonin. Because of tooth extractions and planned jaw lesion irradiation, hypercalcemia was successfully managed with cinacalcet until chemotherapy reduced the PTHrP production by the tumor.

Cinacalcet is a calcimimetic that activates parathyroid CaSRs which are negatively coupled to PTH secretion. Lower PTH levels reduce calcium reabsorption by the kidney, and bone resorption [6]. Activation of renal CaSRs also enhance calcium excretion. Several case reports support the use of cinacalcet in treating PTHrP-mediated hypercalcemia. Bech described a patient with squamous cell carcinoma of the lung with PTHrP-mediated hypercalcemia whose serum calcium declined with cinacalcet treatment. However, chemotherapy was given concurrently with cinacalcet, and PTHrP levels also decreased suggesting the hypothesis that cinacalcet might decrease calcium by decreasing PTHrP secretion by the tumor [7].

Paradoxically, in several in vitro models of PTHrP secreting tumors, increasing CaSR signaling increased PTHrP secretion [8, 9]. In fact, most case studies have not noted dramatic changes in PTHrP with cinacalcet treatment (Table 1). Sheehan reported an 81 year-old woman with non-small cell lung cancer and bladder cancer with hypercalcemia refractory to pamidronate. Cinacalcet was titrated to 60 mg orally twice a day resulting in a reduced serum calcium despite her PTHrP remaining elevated [10]. Sternlicht reported a patient with metastatic renal cell carcinoma who, despite fluids, calcitonin, and a bisphosphonate, remained hypercalcemic at 14.2 mg/dL. After ten weeks of cinacalcet treatment, calcium levels decreased to 10.1 mg/dL while her PTHrP remained elevated [11].

**Table 1:** Case reports and pertinent lab values

Authors	PTHrP before cinacalcet	PTHrP after cinacalcet	1,25 Vitamin D before cinacalcet	1,25 Vitamin D after cinacalcet	Cinacalcet daily dose range
Bech et al. (2012) [7]	13.2 pmol/L (nl < 0.6)	0.9 pmol/L	Not reported	Not reported	60–120 mg
Glezerman et al. (2015) [11]	114 pmol/L (nl 14–27)	159 pmol/L	Not reported	Not reported	60 mg
Asonitis et al. (2017) [12]	Not reported	Not reported	60 pmol/L (nl 18–80)	Not reported	60–90 mg
Valdes-Socin et al. (2017) [13]	Not reported	Not reported	100 pg/mL (nl < 85)	Normalized	120 mg
Sheehan et al. (2020) [10]	41.9 pmol/L (nl < 2.0)	Not reported	Not reported	Not reported	60–120 mg
Sheehan et al. (case 1) (2020) [14]	5.5 pmol/L (< 4.2)	7.8–9.3 pmol/L	104 pg/mL (nl 18–64)	Not reported	60–120 mg
Sheehan et al. (case 2) (2020) [14]	1.2 pmol/L (< 4.2 pmol/mL)	Not reported	55 pg/mL (nl 18–64)	Variable, also was given glucocorticoids	60–120 mg
O’ Callaghan et al. (2021) [15]	47 ng/L (nl 14–27)	32 ng/L	238 pmol/L (nl 43–173)	178 pmol/L	30–120 mg
Christensen et al. (2022) [16]	25 pmol/L (nl < 2.5)	Not reported	513 pg/mL (nl 18–64)	Not reported	Not reported

Other cases of cinacalcet being used in PTHrP-mediated hypercalcemia have been described, but did not report PTHrP levels after cinacalcet treatment. O’Callaghan reported a 71-year-old with hypercalcemia of malignancy initially controlled with zoledronic acid and denosumab. He had a suppressed PTH and a mildly elevated PTHrP. Cinacalcet was started at 30 mg twice a day and eventually titrated to 60 mg twice a day resulting in normal serum calcium levels until he succumbed to an acute stroke 10 months later [15]. In a case of presumed PTHrP-induced hypercalcemia from metastatic breast cancer, Asonitis reported that treatment with cinacalcet decreased serum calcium levels from 13.2 mg/dL to 9.6 mg/dL in one month. Two weeks after stopping cinacalcet therapy, serum calcium rebounded to 10.8 mg/dL. As a proof of concept, cinacalcet was restarted, and two weeks later, calcium decreased to 9.2 mg/dL [12].

There have been two cases of cinacalcet used successfully in patients where both PTHrP and 1,25-dihydroxyvitamin D levels were elevated – one reported by Christensen [16] and the other by Sheehan [14]. Finally, Valdes-Socin reported a case of hypercalcemia associated with a pancreatic neuroendocrine tumor that had an increased 1,25-dihydroxyvitamin D level and was responsive to cinacalcet, but PTHrP was not measured [13]. Of note, there is also a case report of hypercalcemia associated with 1,25-dihydroxyvitamin D elevation but with normal PTHrP that responded to cinacalcet treatment [14].

In all cases, cinacalcet was used in the doses we initiated, and that was followed with improvement in serum calcium within 1 week after dose escalation. Our case is consistent with these prior reports – showing that cinacalcet can reduce hypercalcemia downstream of PTHrP. In fact, on HD 23, PTHrP remained elevated at 7.4 pg/L while the albumin-corrected serum calcium was frankly low. Instead, the dramatic reduction in PTHrP occurred after cinacalcet was discontinued.

Our case is unique from those previously reported in that our patient did not have any prior antiresorptive therapy, suggesting that cinacalcet can be considered as a temporizing measure instead of antiresorptive therapy, in patients at high risk for osteonecrosis of the jaw, or in the setting of renal insufficiency, or if denosumab is not available. The rapid reduction in PTHrP after chemotherapy and attendant hypocalcemia illustrates another advantage of cinacalcet over bisphosphonates or denosumab for the treatment of hypercalcemia. The short half-life of cinacalcet and its short time of action allow for rapid adjustments, while the effects of a bisphosphonate or denosumab are longer lasting, so once given, they are not easily adjusted. Still, there are no data to suggest that skeletal-related events like fractures or bone pain, in the setting of malignancy, can be prevented by cinacalcet.

The other unique contribution of our case is showing that cinacalcet increased urinary calcium excretion. We propose that this is due to activation of renal CaSRs. CaSRs are most highly expressed in the thick ascending limb of Henle. Here, CASRs inhibit the NKCC2 sodium–potassium–chloride cotransporter and the ROMK channel, thereby increasing urinary calcium excretion [17]. Therefore, lasix, which also inhibits NKCC2, may have synergized with cinacalcet. In addition to acting at the apical membrane, CaSR also controls the expression of claudin-14 and claudin-16 at the tight junctions of the thick ascending limb, further inhibiting calcium reabsorption in the kidney [17]. Increased fractional excretion of calcium was not seen in one other case of PTHrP-mediated hypercalcemia after cinacalcet treatment leading to the hypothesis that the effects are mediated by reduced intestinal absorption of calcium [14]. However, this patient also had high levels of 1,25-dihydroxyvitamin D, leaving open the possibility of a different mechanism of action than in pure PTHrP-mediated hypercalcemia. Though we did find an increase fractional excretion of calcium, direct effects on the bone and the gut cannot be ruled out [18]. Unfortunately, we did not measure biochemical markers of bone turnover in this patient before or after cinacalcet treatment. However, the fact that cinacalcet has been effective in cases where repeated doses of denosumab and zoledronic acid have already been given suggests that an effect on osteoclasts is not necessary. Finally, we note that cinacalcet is metabolized by hepatic cytochrome P450 enzymes. Cinacalcet dosing instructions for Child-Pugh Class B patients do not provide specific adjustments, however they do state that drug exposure and half-life for this group are increased. Therefore, the effect of cinacalcet in this patient may have been heightened [19].

Lastly, steroids have been reported to be beneficial in improving hypercalcemia in some cases. These cases are often patients with granulomatous diseases causing hypercalcemia from enhanced intestinal calcium absorption due to increased endogenous calcitriol production. By decreasing calcitriol production, steroids have been shown to thereby reduce serum calcium concentrations. Because our patient had PTHrp-mediated hypercalcemia with a suppressed 1,25 Vitamin D level consistent with this diagnosis, steroids would not likely have benefited him and therefore were not offered.

Of importance with any therapy in an individual with so many co-morbidities, the treatment was well-received and tolerated by the patient, especially given limitations of other agents. He reported ease of administration, which was important given his oral comorbidities, and convenient dosing with his other medications without added burden. He denied any adverse effects of cinacalcet, and overall, he tolerated the treatment well. He was eager to share this case and treatment in hopes of helping future patients. Taken together, we suggest that cinacalcet may be useful for the treatment of PTHrP-mediated hypercalcemia of malignancy especially when the patient is at higher risk for osteonecrosis of the jaw.

## Data Availability

All data generated or analyzed during this study are included in this published article.

## Abbreviations

PTH: Parathyroid hormone
PTHrP: Parathyroid hormone related protein
CaSR: Calcium sensing receptor
